# Identifying impairments and compensatory strategies for temporal gait asymmetry in post-stroke persons

**DOI:** 10.1038/s41598-025-86167-9

**Published:** 2025-01-21

**Authors:** Naomichi Mizuta, Naruhito Hasui, Yasutaka Higa, Ayaka Matsunaga, Sora Ohnishi, Yuki Sato, Tomoki Nakatani, Junji Taguchi, Shu Morioka

**Affiliations:** 1 Department of Rehabilitation, Faculty of Health Sciences, Nihon Fukushi University, 26-2 Higashihaemi-Cho, Handa, Aichi 475-0012 Japan; 2 Neurorehabilitation Research Center, Kio University, 4-2-2 Umaminaka, Kitakatsuragi-gun, Koryo, Nara 635-0832 Japan; 3 Department of Therapy, Takarazuka Rehabilitation Hospital, Takarazuka, Japan; 4 Department of Neurorehabilitation, Graduate School of Health Sciences, Kio University, Koryo, Japan; 5 Department of Medical, Takarazuka Rehabilitation Hospital, Takarazuka, Japan; 6 Graduate School of Frontier Biosciences, Osaka University, Osaka, Japan

**Keywords:** Stroke, Gait, Asymmetry, Compensatory strategies, Neurological disorders, Neurological disorders, Motor control

## Abstract

In post-stroke persons, temporal gait asymmetry (TGA) during comfortable gait involves a combination of pure impairments and compensatory strategies. In this study, we aimed to differentiate between pure impairments and compensatory strategies underlying TGA in post-stroke individuals and identify associated clinical factors. We examined 39 post-stroke individuals who participated in comfortable walking speed (CWS) and rhythmic auditory cueing (RAC). Gait evaluation included spatiotemporal parameters and trunk acceleration, while clinical evaluation comprised motor paralysis severity, sensory disturbances, spasticity, balance ability, and gait efficacy. The participants were classified by clustering based on the symmetry index during CWS and RAC. TGA during CWS or RAC gait showed no association (ρ = 0.062, *p* = 0.707). Clustering yielded four optimal clusters. Cluster 1 was asymmetric during CWS but symmetric during RAC condition (over-compensatory strategies); these participants had a poor score on the modified gait efficacy scale, and despite mild functional impairments, they lacked the confidence to walk safely. Cluster 2 showed impairment-driven asymmetry during CWS and RAC, with severe impairments and instability. Participants showing improved TGA under auditory cueing compared with comfortable speed demonstrate strong compensatory strategies associated with low gait efficacy. This suggests a need for targeted interventions to enhance gait self-efficacy and maximize residual function.

**Supplementary Information:**

The online version contains supplementary material available at 10.1038/s41598-025-86167-9.

## Introduction

Temporal gait asymmetry (TGA) is prevalent in over 50% of post-stroke persons and is widely experienced during inpatient rehabilitation^1,2^. TGA is a condition wherein temporal variables during gait, such as stance and swing times, differ between the paretic and non-paralytic limbs. Post-stroke gait asymmetry has been linked to predicting fall risk and rehabilitation duration^3,4^. A deep understanding of its mechanisms and implications is essential for developing targeted rehabilitation strategies.

Post-stroke gait asymmetry is strongly associated with motor paralysis severity, sensory deficits, and spasticity^5,6^, along with gait instability and psychological factors like confidence in safe gait^7^. Notably, a preference for conservative gait strategies for issues such as fear of falling is also associated with gait asymmetry^8,9^. We consider that “pure impairment” and “compensation strategies” to be involved in post-stroke gait asymmetry. Pure impairment is a situation in which neurological injury caused by stroke, such as motor paralysis, sensory impairment, or spasticity, directly leads to gait asymmetry. In this case, even if the patient consciously tries to modify their gait pattern, it is difficult to achieve a symmetrical gait due to neurological limitations. In contrast, compensatory strategies are gait patterns that patients adopt either consciously or unconsciously to compensate for neurological injury or ensure gait stability. Therefore, some asymmetries may be the result of compensatory strategies. A specific example is the compensatory strategy of increasing the loading time on the non-paretic side due to a lack of confidence in loading on the paretic side. Therefore, given the pure impairment or compensation strategies associated with post-stroke gait asymmetry, deciphering the pathophysiology becomes especially difficult. Post-stroke gait asymmetry is modulated immediately by rhythmic auditory cueing (RAC)^10^. RAC can improve TGA drastically when the patient synchronizes the timing of their foot contact to the tempo of a metronome and can be used as a condition setting to manipulate temporal symmetry during gait^10,11^. However, a previous study investigating temporal asymmetry during comfortable walking speed (CWS) and RAC conditions reported that some cases were asymmetrical in CWS and RAC. In contrast, others were asymmetrical in CWS but symmetrical in RAC^12^. These contrasting cases likely represent a difference in pure impairments or compensatory strategies in TGA. Pure impairment in TGA means that symmetrical gait is not feasible due to severe functional impairment, and cases with a dominance of compensatory strategy may have asymmetrical gait during CWS despite a symmetric gait pattern during RAC. Thus, although TGA includes aspects of pure impairments and compensatory strategies, previous studies did not analyze these separately^2,3,5,13,14^. Intervention strategies for TGA may need to be tailored to the patient’s need based on whether it is predominantly driven by pure impairments or compensatory mechanisms. We categorized TGA in post-stroke persons into two groups: those with pure impairment and those with a predominance of compensatory strategies. The clinical and gait characteristics of these groups were examined. TGA was analyzed under two conditions: CWS and RAC. The subgroups were classified based on symmetry indexes in these conditions using Mixed Gaussian clustering. We assumed that the identified clusters were characterized by motor paralysis, spasticity, various functional impairment severities, and gait self-efficacy. These findings are useful in creating personalized rehabilitation strategies.

## Results

### GMM-based cluster analysis

**Fig. 1:**
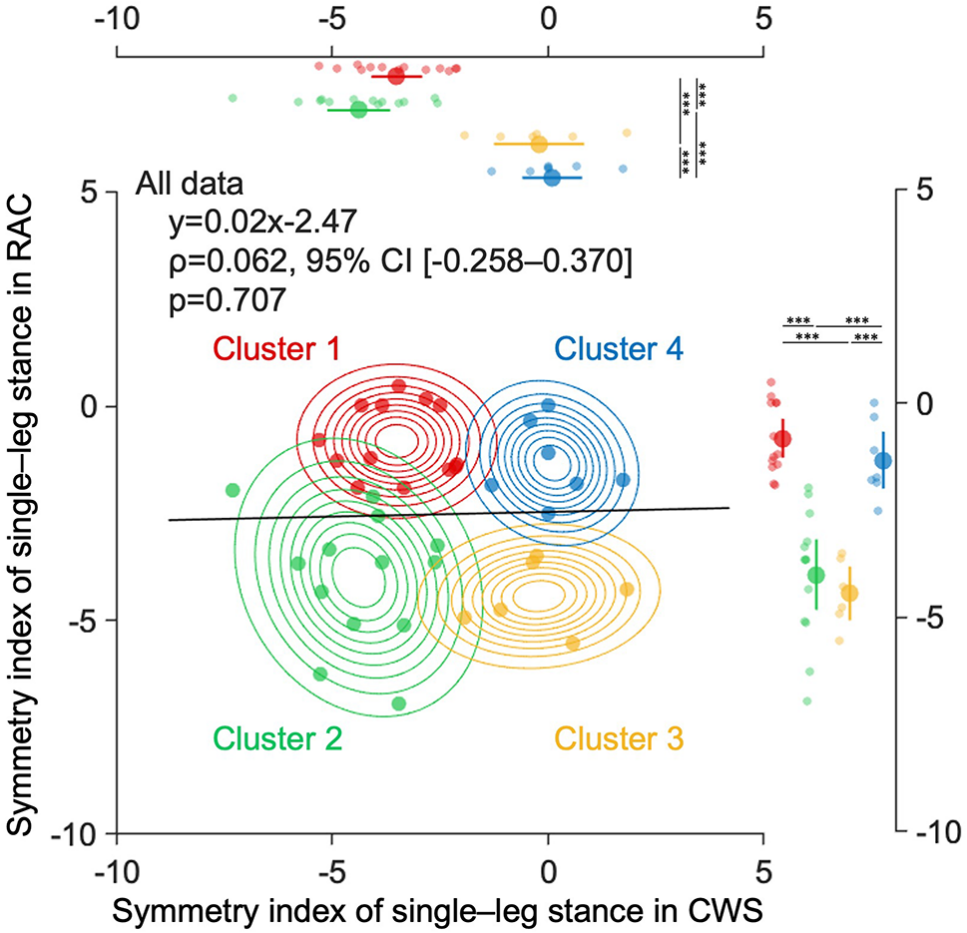
Summary of the Gaussian mixture model-based cluster analysis. Scatterplots of the symmetry index of single–leg stance time during gait in CWS and RAC conditions for each cluster based on mixed Gaussian clustering and the outermost 95% confidence ellipsoid. The black line represents the regression line for all data. The symmetry index in the CWS and RAC conditions scatterplots and 95% confidence ellipses show the independence and characteristics of the four clusters. The top and right plots show the mean of the symmetry index of single–leg stance time during gait in CWS and RAC conditions, the 95% confidence interval, and each data point for each cluster. The negative symmetry index represents longer single–leg stance time on the non-paretic side. Statistical significance was determined using post-hoc testing (Steel–Dwass test) and the Kruskal–Wallis test, denoted by ***P < 0.001. *CWS* comfortable walking speed,* RAC* rhythmic auditory cueing.

No association was found between the symmetry indexes in the CWS and RAC conditions (ρ = 0.062, 95% confidence interval [-0.258–0.370], *p* = 0.707), and the scatter plots of these two parameters showed that the symmetry indexes were low and high in the CWS and RAC conditions, respectively. Therefore, GMM-based clustering was performed using these two variables to classify the subgroups based on the distribution of symmetry indexes in the CWS and RAC conditions. Of the several models that satisfied these criteria, we selected one that showed higher theoretical validity with better Bayesian information criterion (BIC) and integrated complete-data likelihood (ICL) as the optimal model (Table S1). Four optimal clusters were identified from the distribution of symmetry indexes in the CWS and RAC conditions. Figure 1 shows a scatterplot of the symmetry indexes in the CWS and RAC conditions for each cluster, with the outermost ellipse being the 95% confidence level of the distribution, indicating each cluster’s independence and characteristics. Clusters 1 and 3 were represented by ellipses with high variability of the symmetry index in the CWS condition. In contrast, the variability of the symmetry index in the RAC condition was relatively small. Clusters 2 and 4 were represented by ellipses with a large variance in the symmetry index under the RAC condition. The symmetry index in the CWS condition differed among the clusters (χ2 = 26.690, df = 3, ε2 = 0.702, *p* < 0.001), with that of Clusters 1 and 2 being significantly lower than that of 3 and 4. The symmetry index in the RAC condition also varied among the Clusters (χ2 = 28.521, df = 3, ε2 = 0.751, *p* < 0.001), with that of Clusters 2 and 3 being significantly lower than that of 1 and 4 (Fig. 1).

These results indicate that Cluster 1 signified asymmetric gait during CWS with the ability to walk symmetrically with RAC, whereas Cluster 2 signified asymmetric gait during CWS and the inability to walk symmetrically even with RAC.

### Clinical and gait characteristics

We conducted a repeated-measures two-way analysis of variance (RM-ANOVA) to confirm whether gait speed and cadence were controlled under the two conditions. Gait speed in the CWS and RAC conditions showed a main effect of cluster (F(3,35) = 0.547, *p* = 0.002, η_p_^2^ = 0.334) and no main effect of condition (F(1,35) = 0.916, *p* = 0.345, η_p_^2^ = 0.026) and interaction (F(3,35) = 1.960, *p* = 0.138, η_p_^2^ = 0.144). Cadence did not have a main effect of cluster (F(3,35) = 1.696, *p* = 0.186, η_p_^2^ = 0.127) or condition (F(1,35) = 0.277, *p* = 0.602, η_p_^2^ = 0.008), nor an interaction effect (F(3,35) = 1.034, *p* = 0.389, η_p_^2^ = 0.081). The absence of a main effect or interaction between gait speed and cadence during gait conditions indicates that the spatiotemporal parameters of gait were controlled in the CWS and RAC conditions. In contrast, there was no main effect of condition (F(3,35) = 1.177, *p* = 0.285, η_p_^2^ = 0.033) for the single-leg stance time on the paretic side, but a main effect of cluster (F(3,35) = 6.113, *p* = 0.002, η_p_^2^ = 0.344) and an interaction (F(3,35) = 15.29, *p* < 0.001, η_p_^2^ = 0.567) were observed.

**Fig. 2:**
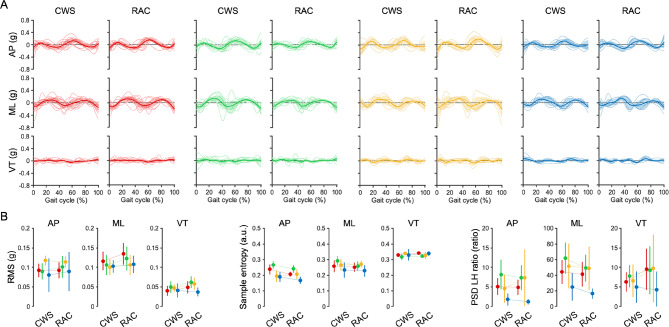
Time-series signals and features of trunk acceleration for each cluster between gait conditions. (**A**) Time series signals of trunk acceleration among clusters based on the gait conditions. Red, Cluster 1; green, Cluster 2; yellow, Cluster 3; blue, Cluster 4. Deep lines represent the means, area plots represent 95% confidence intervals, and thin lines represent the plots of individual participants within a cluster. (**B**) The RMS, sample entropy, and PSD LH ratio of the trunk acceleration signal during the entire gait cycle. Higher values of RMS and sample entropy indicate instability, while higher values of PSD LH ratio indicate a larger PSD component in the 0.5–3 Hz range. The plot included means and 95% confidence intervals (CIs).* CWS* comfortable walking speed,* RAC* rhythmic auditory cueing,* AP* anteroposterior,* ML* mediolateral,* VT* vertical,* RMS* root mean square,* PSD* power spectral density, * LH ratio* low-high ratio.

Figure 2 summarizes the clinical and gait evaluation results for each cluster, and Table 1 lists the demographic characteristics. Notably, each cluster yielded different results for each evaluation. Cluster 2 had lower Fugl–Meyer Assessment (FMA) synergy score (FMS) and trunk impairment scale scores, but the FMA sensory score and Short-Form Berg Balance Scale scores did not differ among the clusters. The Modified Ashworth Scale (MAS) was higher in Cluster 2, and the modified Gait Efficacy Scale (mGES) score was lower in Clusters 1 and 2. Gait speed was lower in Cluster 2; however, cadence did not differ. These results suggest that, among Clusters 1 and 2, where the symmetry index was low in the CWS condition, Cluster 2 was characterized by a dependence on functional impairment, such as motor paralysis and poor spasticity scores. In contrast, Cluster 1 was characterized by asymmetry during CWS, even without significant functional impairment. Cluster 1 was characterized by asymmetry in CWS without significant dysfunction, low values on the modified gait efficacy scale, and low confidence in gait safety (Table 2). Figure S1 shows the associations between the clinical evaluations.

**Table 1:** Characteristics of each cluster.

	All participants	Cluster 1 (*n* = 13)	Cluster 2 (*n* = 13)	Cluster 3 (*n* = 6)	Cluster 4 (*n* = 7)
Age (years)^a^	70.4 ± 10.7	75.6 ± 10.8	70.5 ± 9.89	66.3 ± 10.2	63.9 ± 9.05
Time since stroke (days)^a^	78.8 ± 42.6	79.3 ± 51.9	77.8 ± 43.1	64.8 ± 14.3	91.6 ± 42.8
Sex: male/female^b^	23/16	8/5	8/5	3/3	4/3
Affect side: right/left^b^	24/15	8/5	9/4	3/3	4/3
Functional ambulation category^c^	3 (3 − 4)	3 (3 − 4)	3 (3 − 4)	3.5 (3 − 4)	4 (4 − 5)
Using assist device: no use / T-cane / Q-cane^b^	34/ 5/0	12/1/0	11/2/0	5/1/0	6/1/0
Fugl − Meyer assessment synergy score^c^	22 (21 − 22)	22 (21 − 22)	20 (19 − 21)	22 (22 − 22)	22 (22 − 22)
Fugl − Meyer assessment sensory^c^	10 (10 − 12)	10 (9 − 12)	10 (9 − 12)	12 (10.5 − 12)	12 (10 − 12)
Modified Ashworth score^c^	0 (0 − 1)	0 (0 − 0)	1 (1 − 2)	0 (0 − 0)	0 (0 − 0)
Trunk impairment scale^c^	19 (17.5 − 21.5)	19 (19 − 21)	17 (15 − 18)	21.5 (19.25 − 23)	20 (19 − 23)
Short − form Berg balance scale^c^	23 (20 − 25)	22 (20 − 25)	22 (20 − 25)	22 (19.25 − 24.75)	25 (24 − 28)
Modified gait efficacy scale^c^	18 (13.5 − 24)	14 (13 − 17)	16 (13 − 20)	25 (23 − 27)	27 (27 − 28)

^a^Mean ± standard deviation. ^b^Absolute number. ^c^Median (interquartile range).

**Table 2:** Comparison of clinical evaluations among each cluster.

Steel–Dwass	FMA synergy score		FMA sensory score		Modified Ashworth scale		Trunk impairment scale		Short–form Berg balance scale		Modified gait efficacy scale	
Cluster	W	p-value	W	p-value	W	p-value	W	p-value	W	p-value	W	p-value
1 vs. 2	− 3.612	0.052	0.038	1.000	2.900	0.170	− 3.433	0.072	− 0.185	0.999	2.120	0.438
1 vs. 3	1.371	0.767	2.229	0.393	− 1.759	0.599	1.845	0.560	− 0.063	1.000	4.545	0.007**
1 vs. 4	1.607	0.667	1.646	0.650	− 1.895	0.538	1.502	0.713	2.273	0.375	4.675	0.005**
2 vs. 3	3.561	0.057	2.003	0.489	− 3.993	0.025*	3.886	0.031*	0.126	1.000	3.675	0.046*
2 vs. 4	3.834	0.034*	1.432	0.742	− 4.246	0.014*	3.967	0.026*	2.670	0.233	4.344	0.012*
3 vs. 4	0.161	1.000	− 0.704	0.960	N/A	N/A	− 0.439	0.990	1.660	0.644	1.584	0.677

The p-values were determined using the post-hoc tests (Steel–Dwass) for the clinical and gait evaluations of each cluster. The Modified Ashworth scale between clusters 3 and 4 was indicated as N/A since the scores of all participants in these clusters were the same. *FMA* Fugl–Meyer Assessment. *P < 0.05, **P < 0.01.

The trunk acceleration variables for the clusters across gait conditions had several characteristics (Fig. 2): the anterior-posterior components of trunk acceleration in the CWS and RAC conditions showed no main effects of cluster (F(3,35) = 0.661, *p* = 0.376, η_p_^2^ = 0.002) or condition (F(1,35) = 1.643, *p* = 0.209, η_p_^2^ = 0.049), but an interaction effect (F(3,35) = 3.223, *p* = 0.035, η_p_^2^ = 0.232), while Clusters 1 and 2 showed a simple main effect of condition. This indicates that trunk sway increased during the RAC condition only in Clusters 1 and 2. Only a main effect of condition was observed for the mediolateral components of trunk acceleration (F(1,35) = 12.280, *p* = 0.001, η_p_^2^ = 0.227). The detailed results are presented in Table 3. Sample entropy showed a main effect of cluster (F(3,35) = 6.423, *p* = 0.002, η_p_^2^ = 0.376) and condition (F(1,35) = 4.815, *p* = 0.036, η_p_^2^ = 0.131) only for the anterior-posterior component, but no interaction effect was observed (F(3,35) = 1.697, *p* = 0.187, η_p_^2^ = 0.137). This indicates that the sample entropy of the anterior-posterior component was higher in Clusters 1 and 2 during the CWS condition, and the gait was less regular. In addition, only Cluster 3 showed an increase in the anterior-posterior component under the RAC condition. The Welch power spectral density (PSD) low-high (LH) ratio showed a main effect of condition only for the mediolateral component (F(1,35) = 7.223, *p* = 0.011, η_p_^2^ = 0.184). This result indicates that the PSD ratio in the 0.5–3 Hz band (Low) was lower than that in the 3–10 Hz band (High) in the RAC condition compared to the CWS condition, suggesting that the gait motion exhibited small incremental frequency components.

**Table 3:** Repeated-measures two-way ANOVA of gait parameters across gait conditions and clusters.

		Cluster 1		Cluster 2		Cluster 3		Cluster 4		Main effects				Interaction	
										Conditions		Cluster		Condition x cluster	
		CWS	RAC	CWS	RAC	CWS	RAC	CWS	RAC	p	ηp2	p	ηp^2^	p	ηp^2^
Gait speed		0.919 (0.802−1.037)	0.888 (0.769−1.006)	0.772 (0.687−0.856)	0.796 (0.705−0.887)	0.980 (0.788−1.171)	0.980 (0.684−1.276)	1.170 (0.984−1.356)	1.249 (1.063−1.435)	0.262	0.036	**0.002**	0.338	0.098	0.162
Cadence		110.4 (104.9−115.9)	109.5 (103.4−115.7)	108.2 (103.8−112.5)	110.5 (105.6−115.3)	108.2 (95.3−121.0)	109.0 (93.2−124.8)	120.2 (111.1−129.3)	119.7 (112.2−127.1)	0.602	0.008	0.186	0.127	0.389	0.081
Single-leg stance time		36.8 (35.3−38.2)	40.1 (38.7−41.5)	35.9 (35.2−36.6)	37.2 (35.6−38.8)	41.5 (38.4−44.6)	36.0 (31.2−40.8)	41.3 (38.9−43.7)	40.1 (38.4−41.9)	0.285	0.033	**0.002**	0.344	**< 0.001**	0.567
Symmetry index		– 3.509 (– 4.161−– 2.857)	– 0.822 (– 1.329−– 0.316)	– 4.391 (– 5.200−– 3.583)	– 4.002 (– 4.917−– 3.088)	– 0.214 (– 1.585−1.157)	– 4.441 (– 5.265−– 3.617)	0.093 (– 0.771−0.956)	– 1.332 (– 2.161−– 0.502)	**0.035**	0.121	**< 0.001**	0.755	**< 0.001**	0.677
RMS	AP	0.100 (0.083−0.117)	0.115 (0.093−0.138)	0.095 (0.075−0.116)	0.107 (0.079−0.135)	0.116 (0.098−0.134)	0.112 (0.087−0.137)	0.093 (0.070−0.117)	0.087 (0.064−0.110)	0.209	0.049	0.613	0.054	**0.035**	0.232
	ML	0.116 (0.092−0.140)	0.135 (0.107−0.162)	0.106 (0.080−0.132)	0.123 (0.092−0.153)	0.100 (0.080−0.121)	0.106 (0.080−0.132)	0.103 (0.086−0.119)	0.108 (0.085−0.130)	**0.001**	0.277	0.626	0.052	0.302	0.106
	VT	0.040 (0.027−0.053)	0.049 (0.033−0.065)	0.050 (0.035−0.064)	0.062 (0.047−0.077)	0.047 (0.038−0.057)	0.058 (0.043−0.073)	0.041 (0.023−0.059)	0.037 (0.026−0.049)	0.121	0.074	0.261	0.116	0.619	0.053
Sample entropy	AP	0.237 (0.205−0.269)	0.206 (0.189−0.222)	0.265 (0.239−0.290)	0.241 (0.215−0.266)	0.193 (0.157−0.229)	0.207 (0.176−0.237)	0.188 (0.157−0.219)	0.167 (0.144−0.191)	**0.036**	0.131	**0.002**	0.376	0.187	0.137
	ML	0.257 (0.219−0.295)	0.251 (0.220−0.282)	0.294 (0.264−0.323)	0.259 (0.229−0.289)	0.266 (0.229−0.303)	0.271 (0.248−0.293)	0.233 (0.183−0.282)	0.228 (0.191−0.265)	0.121	0.074	0.310	0.104	0.127	0.161
	VT	0.331 (0.320−0.342)	0.341 (0.333−0.349)	0.318 (0.296−0.340)	0.322 (0.311−0.332)	0.340 (0.329−0.350)	0.328 (0.305−0.351)	0.330 (0.294−0.367)	0.339 (0.324−0.354)	0.580	0.010	0.352	0.096	0.413	0.084
PSD LH ratio	AP	5.085 (2.934−7.235)	4.896 (2.954−6.838)	8.185 (4.369−12.00)	7.393 (4.205−10.58)	4.552 (0.973−8.131)	7.373 (0.155−14.6)	1.794 (0.254−3.334)	1.241 (0.512−1.971)	0.510	0.014	0.069	0.196	0.080	0.188
	ML	44.2 (28.7−59.7)	41.2 (25.4−57.0)	61.8 (41.9−81.8)	49.0 (31.8−66.2)	51.8 (23.2−80.3)	48.8 (20.9−76.7)	25.0 (7.30−42.6)	16.5 (9.90−23.1)	**0.011**	0.184	0.091	0.181	0.374	0.092
	VT	6.271 (3.802−8.739)	9.525 (4.239−14.8)	7.760 (4.893−10.6)	9.254 (3.105−15.4)	6.573 (2.408−10.7)	9.718 (1.087−18.4)	4.963 (0.967−8.96)	4.252 (– 0.400−8.90)	0.143	0.066	0.650	0.049	0.640	0.051

The data are reported as mean (95% confidence interval). Bolded p-values (p) denote statistical significance at *p* < 0.05. Single-leg stance time indicates the percentage of the gait cycle, and is the value for the paretic side. ηp^2^ is an effect size indicator in a two-way analysis of variance. The symmetry index is calculated based on the single-leg stance time of both legs.* ANOVA* analysis of variance,* CWS* comfortable walking speed,* RAC* rhythmic auditory cueing,* RMS* root mean square,* AP* anteroposterior,* ML* mediolateral,* VT* vertical,* PSD* power spectral density,* LH ratio* low-to-high ratio.

## Discussion

In this study, TGA was experimentally manipulated in post-stroke persons to investigate their impairment and compensatory strategies. Temporal asymmetry during CWS and RAC gait showed no association and varied widely. The clustering of participants based on temporal asymmetry resulted in the identification of four subgroups, each with unique clinical and gait characteristics. Two clusters were characterized by the predominance of pure impairment and compensatory strategies in TGA, which was the primary aim of this study. Notably, participants who exhibited asymmetry during the CWS but achieved symmetrical gait during RAC showed strong compensatory strategies due to decreased gait self-efficacy, suggesting that they did not fully utilize their residual function. Each cluster offers a valuable framework for characterizing these deviations and can serve as a basis for developing tailored intervention strategies for post-stroke individuals. By observing temporal asymmetries during gait, the two components of impairment and compensatory strategies can be effectively resolved by observing temporal asymmetries during gait.

### Characteristic classification of TGA

As expected, TGA varied in post-stroke persons, with no association between the symmetry indexes in the CWS and RAC conditions (Fig. 1). Four distinct clusters were identified based on the symmetry indexes under CWS and RAC, associated with varying clinical and gait characteristics. This highlights the importance of considering individual differences in temporal asymmetry when evaluating post-stroke gait. Notably, some participants showed low symmetry in CWS but high symmetry in RAC, revealing the need to distinguish between impairment and compensatory strategies. Previous studies have measured gait asymmetry during CWS^2,3,5,13,14^. However, since CWS involves pure impairment and compensation strategies, it is essential to establish conditions that can manipulate and differentiate these specific factors. The four-cluster classification in this study enabled us to differentiate between temporal asymmetry caused by pure impairment and that resulting from compensatory strategies. This differentiation is crucial for gaining insights into impaired gait structure and will benefit future research and experimental methods.

### Clinical and gait characteristics for each cluster

Gait asymmetry in post-stroke persons strongly correlates with the severity of motor paralysis, sensory deficits, and spasticity^5,6^. In addition to post-stroke functional impairments, balance ability and confidence in gait safety (fear of falling) are also associated with gait asymmetry^7–9^. Conversely, the clusters based on the temporal symmetry indexes in the CWS and RAC conditions explored in the study exhibited distinct clinical and gait parameter characteristics, particularly Clusters 1 and 2, characterized by pure impairment and compensatory strategies, respectively.

Cluster 2 showed asymmetry in both conditions, and it is thought that it failed to achieve symmetry even with RAC due to severe paretic leg dysfunction and gait instability (Fig. 2B). In fact, TGA is related to functional impairment and gait instability in post-stroke^5–7^. Therefore, we considered that even if the participants in Cluster 2 tried to modify their gait pattern consciously, they would still have difficulty achieving symmetrical gait due to their neurological impairments. The temporal asymmetry of this cluster during CWS reflected these participants’ pure impairment, suggesting that rehabilitation focusing on paretic leg dysfunction and gait instability is beneficial.

In contrast, participants in Cluster 1 exhibited asymmetry in the CWS condition and symmetry in the RAC condition (Fig. 1). Cluster 1 demonstrated mild functional impairment of the paretic leg, similar to Clusters 3 and 4, yet showed temporal asymmetry in the CWS condition. This observation may be interpreted as a compensatory strategy rather than a sign of impairment. Furthermore, Cluster 1 showed low mGES scores and poor self-confidence in gait safety (Table 2). Post-stroke persons with poor gait self-efficacy and low confidence in their balance abilities tend to exhibit altered temporal gait parameters, such as increased double support time, step time variability, and reduced gait speed^8^. Tinetti and Powell defined fear of falling as “a persistent anxiety about falling that results in avoidance of activities that the person would otherwise be able to perform^15^, ” and suggested that activity avoidance could lead to motor limitations, thereby decreasing functional capacity and increasing the risk and fear of falling^8,16,17^. Previous research has revealed that some patients with mild functional impairments post-stroke have strategically altered their gait^18^. We believe that the asymmetry in CWS in Cluster 1 was not due to dependence on the impaired function of the paretic lower limb; rather it was a strategy that prioritized dependence on the non-paretic lower limb based on a decrease in self-efficacy for gait. Gait asymmetry in post-stroke persons has significant implications for critical social outcomes, such as predicting future fall risks and length of rehabilitation^3,4^. These findings suggest that participants in Cluster 1, who experienced mild functional impairment but demonstrated temporal asymmetry during CWS, do not fully utilize their residual function due to a lack of confidence in their ability to walk safely. This results in an over-reliance on compensatory strategies during gain and in daily life. Identifying and characterizing these clusters is crucial for gait maintenance and improvement, as participants in Cluster 1 may benefit from rehabilitation focused on improving gait self-efficacy and reducing fear of falling^19,20^.

Cluster 3 comprised participants who exhibited symmetric gait during the CWS but asymmetric gait during the RAC condition (Fig. 1). The RAC condition required dual-task, as gait must be consciously adjusted to auditory stimulation^21^. Notably, some patients with stroke demonstrate significantly worse gait performance during dual-task activities^22,23^, which is believed to result from impaired automatic control of gait during single-task conditions such as the CWS. Cluster 3 showed an increase in the sample entropy of the anterior-posterior component during the RAC condition (Fig. 2B), while other clusters decreased in the RAC condition. An increase in sample entropy indicates reduced signal regularity, signifying a less stable gait^24,25^. Therefore, we consider that Cluster 3 had impaired automatic gait control during the single task, resulting in less stable and asymmetric gait in the RAC condition.

### Limitations and future directions

The following limitations should be considered when interpreting the results: First, we could not draw causal inferences regarding the relationships between clinical characteristics and gait ability due to the cross-sectional study design. Longitudinal studies are required to clarify these causal relationships. Second, the relatively small sample size may limit the generalizability of our results. Future studies should examine causal relationships in larger samples using more comprehensive measures. Third, the clustering analysis used only temporal symmetry to classify participants into different clusters. These variables are important in determining symmetry and compensatory strategies during gait; however, spatial symmetry variables such as stride length might have contributed to the clustering of participants. Fourth, we focused on three selected items from the mGES rather than utilizing the entire scale. Consequently, the multifaceted evaluation provided by the comprehensive mGES may not be fully represented. The items were selected based on the research objectives; therefore, conclusions drawn should be interpreted from the perspective of these three items.

## Conclusions

Our findings revealed distinct clusters of TGA in post-stroke persons under experimental gait conditions and highlighted the need to decompose TGA into two components: pure impairments and compensatory strategies. These findings offer valuable insights that can facilitate the development of personalized rehabilitation strategies for post-stroke persons, given that the severity of functional impairments and gait self-efficacy differ among clusters. Future research should investigate the clustering of participants using a more comprehensive set of variables to better understand temporal and spatial asymmetry impairments and compensatory mechanisms.

## Methods

### Participants

This cross-sectional study enrolled 39 post-stroke persons aged 70.4 ± 10.7 years (mean ± standard deviation), with stroke onset at 78.8 ± 42.6 d at a rehabilitation hospital. This study adheres to all STROBE guidelines and reports the required information accordingly. The inclusion criteria were participants who could walk independently without the assistance of a physical therapist. The exclusion criteria were as follows: (1) inability to walk without leg braces, (2) bilateral lesions, (3) Mini-Mental State Examination score < 24 points, (4) history of orthopedic disease, (5) pain, (6) cerebellar lesions or resting tremor, and (7) patients with unilateral spatial neglect. We enrolled persons who did not meet the exclusion criteria. Informed consent was obtained from all participants before their involvement in the study. All procedures were approved by the Ethics Committee of Takarazuka Rehabilitation Hospital (ethics review number: 2019-P-2) and conducted in accordance with the Declaration of Helsinki.

### Study design and procedures

All participants were instructed to walk thrice on a 10-m walkway with a supplementary 6-m walkway, as the physical therapist stood nearby to assist if faltering. The participants were allowed to use a cane to prevent falls during the assessments; however, ankle-foot and knee orthoses were not permitted. They were also instructed to walk under two gait conditions: CWS and RAC (walking with rhythmic auditory cueing). In the RAC condition, the tempo was determined by calculating the cadence of the CWS before starting the measurements. Participants listened to a smartphone auditory metronome application (MetroTimer version 3.3.2, ONYX Apps) in the RAC condition. They were asked to match their heel contact timing to the beat of the metronome^12,26^. We instructed the participants to walk at the same speed as the CWS in the RAC. The measurement order for each gait condition was based on a randomized block design. Before recording, a score of < 4 was confirmed on the modified Borg scale to ensure that fatigue did not affect performance. Wireless inertial sensors (Delsys Trigno, Delsys Inc., Boston, MA; sampling rate: 2000 Hz) were attached to the dorsal surface of the third lumbar vertebra and the distal lower leg on both sides to record trunk stability data and the gait cycle of both legs.

### Clinical evaluation

The severity of motor paralysis and sensory disturbances were measured using the FMA. The FMS was used to determine the FMA motor score^27,28^. The response of the ankle plantar flexor muscle was assessed using the MAS converted to a 5-point scale (0, no increase in muscle tone; 5, the affected part is rigid in flexion or extension) to evaluate spasticity. Motor performance was assessed using the trunk impairment scale, the Short-Form Berg Balance Scale, and the Functional Ambulation Category; the self-efficacy of gait was assessed utilizing the mGES. We used the sum of three items (on a 30-point scale) from the mGES−“walking on a flat surface,” “walking on grass,” and “walking a long distance”−to evaluate gait self-efficacy. While the mGES includes items related to stair climbing and curb climbing, we excluded these items from our analysis. This exclusion was deemed necessary as not all hospitalized stroke patients in our study had experience with stair climbing or curb climbing due to them rehabilitation stage. Including these items can have introduced bias into the dataset, potentially confounding the accurate assessment of gait self-efficacy.

### Data recording and analysis

Gait speed and cadence were measured with a stopwatch when the participants walked between the start and end lines of the 10-m walkway. In the following analysis, the first and last three gait cycles were eliminated from the dataset to prevent confounding effects due to acceleration and deceleration. Gait events were identified based on the angular velocity of both inertial sensors to identify the timing of initial contact and toe-off^29,30^. A spline interpolation for 20 gait cycles based on the timing of the initial contact was adapted to fit the inertial signals to a normalized 201-point gait cycle time base.

The symmetry of the single-leg stance time was evaluated using the symmetry index^31,32^. The symmetry index was calculated as follows:

$$\:SI\:=\:\frac{\left({X}_{p}-{X}_{np}\right)}{0.5\left({X}_{p}+{X}_{np}\right)}\times\:100\%$$

where *Xp* and *Xnp* are the values of the gait variable measured for the paretic and non-paretic sides, respectively. When *SI* = 0, the gait is symmetrical.

The accelerations from a tri-axial inertial sensor attached to the third lumbar level were obtained and^33^, as previously described, the subtracted mean value was subjected to a low-pass filter using a zero-lag 4th -order Butterworth filter at a cutoff frequency of 10 Hz to quantify trunk instability, and the root mean square waveform was obtained. Since the acceleration value is proportional to the square of the velocity, the acceleration signal was corrected by dividing it by the squared value of gait speed^33^.

Sample entropy (SampEn) is a robust measure of time-series regularity, reflecting the predictability and complexity of a system. Lower SampEn values indicate more predictable and stable patterns, while higher values suggest greater irregularity and unpredictability^34^. We employed the SampEn (m, r, N) algorithm developed by Richman and Moorman to quantify the regularity of trunk acceleration signals during gait^34^. This algorithm first defines a template of length “m”, representing a specific sequence of “m” consecutive data points within the time series of trunk acceleration data. The algorithm then searches for similar templates within the time series that match the initial template within a predefined tolerance “r”, representing the allowed difference between corresponding data points. This match-counting process was repeated over the time series to compute the final output, as shown in the following equation:

$$\:SampEn\:=\:-In\left(\frac{\sum\:Ai}{\sum\:{B}_{i}}\right)$$

where *Ai* is the count of matches of length “*m* + 1” with the *i*th template, and *Bi* is the count of matches of length *“m”* with the *i*th template.

Power spectral density (PSD) analysis was conducted using Welch’s method to extract detailed kinematic information regarding trunk control during gait embedded within the trunk acceleration data^35^. Specifically, the trunk acceleration data, acquired at a sampling rate of 2000 Hz, were segmented using a 1000-ms Hamming window with a 500 ms overlap, followed by a Fast Fourier Transform (FFT). Combining Hamming windowing and overlapping effectively minimized spectral leakage while achieving a frequency resolution of 0.061 Hz, which provided sufficient granularity to detect subtle features within the gait cycle. Based on the primary frequency bands associated with trunk sway during gait as defined in previous research^35^, the resulting PSD was divided into a low-frequency band (0.5–3 Hz) and a high-frequency band (3–10 Hz). The mean power in each frequency band was calculated, and the ratio of low-frequency to high-frequency power (PSD low-high ratio: LH ratio) was computed.

### Gaussian mixture model clustering for limb kinematics characteristics

This study used a statistical method based on data distribution to avoid subjective decisions when analyzing the complex structure of asymmetry during gait. In this study, the symmetry index of single-leg stance time in CWS and RAC conditions was used to classify participants using Gaussian mixture model (GMM)-based clustering. This procedure allowed us to understand the characteristics of asymmetry during CWS and RAC for participants. Two criteria for the number of clusters and distribution features were defined: (1) the number of clusters was between four and seven (for a more detailed classification than the four categories of high/low symmetry index of single-leg stance time in the CWS condition and high/low symmetry index of single-leg stance time in the RAC condition), and (2) the model distribution parameters were not equally distributed. Of the several computed models, the optimal model(s) selected was the one with good BIC and ICL values, along with high theoretical validity. MATLAB R2019b (MathWorks, Natick, MA) was used for all analyses.

### Statistical analyses

Normality was assessed using the Shapiro–Wilk test and the variables were log-transformed as needed. The differences in basic attributes, clinical test scores, and gait parameters in each cluster were evaluated using the chi-squared and Kruskal–Wallis tests (with a post-hoc Steel–Dwass test). To confirm the effect of the gait condition among clusters, a 2 × 4 RM-ANOVA [Condition (CWS/RAC) × Cluster (1/2/3/4)] was conducted to examine the gait parameters. A priori power analysis using G*Power (version 3.1.9.6) indicated that a total sample size of 32 participants was required to achieve a power of 0.80 with an effect size of ηp² = 0.45, alpha level of 0.05, and a correlation of 0.062 between repeated measures. Partial eta squared (ηp²) values were computed to assess the effect size. A typical interpretation classifies the effect size as small (ηp² = 0.01), medium (ηp² = 0.06), or large (ηp² = 0.14)^36^. Spearman’s rank correlation coefficient was used to confirm all the parameters. Statistical significance was set at *p* < 0.05, and all statistical analyses were performed using R version 4.1.0 (R Foundation, Vienna, Austria).

## Electronic supplementary material

Below is the link to the electronic supplementary material.

Supplementary Material 1

## Data Availability

The data used in this study are available on request, in anonymized format, from the corresponding author.
