# Supplementary material for: Identifying impairments and compensatory strategies for temporal gait asymmetry in post-stroke persons
Source: Sci Rep. 2025 Jan 21;15:2704. doi: 10.1038/s41598-025-86167-9 (PMC11751082; doi:10.1038/s41598-025-86167-9)
Supplement: Supplementary file 1 — Supplementary Material 1 [file 41598_2025_86167_MOESM1_ESM.docx]

**Supplementary Material**

Identifying Impairments and Compensatory Strategies for Temporal Gait Asymmetry in Post-stroke Persons

**Naomichi Mizuta, PT, PhD ^a,b*^, Naruhito Hasui, PT, MS ^c,d^, Yasutaka Higa, PT ^c^, Ayaka Matsunaga, PT ^c^, Sora Ohnishi, PT ^c,d^, Yuki Sato, PT, MS ^f^, Tomoki Nakatani, PT ^c^, Junji Taguchi, MD, PhD ^e^, Shu Morioka, PT, PhD ^b,d*^**

^a^Department of Rehabilitation, Faculty of Health Sciences, Nihon Fukushi University, Handa, Japan

^b^Neurorehabilitation Research Center, Kio University, Koryo, Japan

^c^Department of Therapy, Takarazuka Rehabilitation Hospital, Takarazuka, Japan

^d^Department of Neurorehabilitation, Graduate School of Health Sciences, Kio University, Koryo, Japan

^e^Department of Medical, Takarazuka Rehabilitation Hospital, Takarazuka, Japan

^f^Graduate School of Frontier Biosciences, Osaka University, Osaka, Japan

***Corresponding authors:**

Dr. Naomichi Mizuta

Department of Rehabilitation, Faculty of Health Sciences, Nihon Fukushi University

26-2 Higashihaemi-Cho, Handa-Shi, Aichi 475-0012, Japan

Tel.: +81 569-20-0111; Fax.: +81 569-20-0119

Email: mizuta-n@n-fukushi.ac.jp

**ORCID:** 0000-0002-6177-6967

Dr. Shu Morioka

Neurorehabilitation Research Center, Kio University

4-2-2 Umaminaka, Koryo, Kitakatsuragi-gun, Nara 635-0832, Japan

Tel.: +81 745-54-1601; Fax.: +81 745-54-1600​

Email: s.morioka@kio.ac.jp

**ORCID:** 0000-0003-2853-1789

**Fig S1. Correlation of clinical evaluation**

FMS, Fugl–Meyer Assessment Synergy; FMA Sensory, Fugl–Meyer Assessment Sensory; MAS, Modified Ashworth Scale; TIS, trunk impairment scale; SFBBS, Short–form Berg Balance Scale; FAC, Functional Ambulation Category; mGES, modified Gait Efficacy Scale.

**Table S1. Criteria for selecting the number of clusters.**

| Number of clusters | Bayesian information criterion | Integrated complete data likelihood |
| --- | --- | --- |
| 4 | 410.3 | 141.5 |
| 5 | 411.2 | 143.3 |
| 6 | 412.5 | 144.0 |
| 7 | 413.9 | 144.7 |
